# Potency assays and biomarkers for cell-based advanced therapy medicinal products

**DOI:** 10.3389/fimmu.2023.1186224

**Published:** 2023-06-09

**Authors:** Chiara Capelli, Carolina Cuofano, Chiara Pavoni, Simona Frigerio, Daniela Lisini, Sara Nava, Michele Quaroni, Valentina Colombo, Francesco Galli, Svetlana Bezukladova, Paola Panina-Bordignon, Giuseppe Gaipa, Patrizia Comoli, Giulio Cossu, Gianvito Martino, Andrea Biondi, Martino Introna, Josée Golay

**Affiliations:** ^1^ Center of Cellular Therapy “G. Lanzani”, ASST Papa Giovanni XXIII, Bergamo, Italy; ^2^ Cell Therapy Production Unit, Fondazione IRCCS Istituto Neurologico Carlo Besta, Milan, Italy; ^3^ Laboratory of Cell and Gene Therapy Stefano Verri, ASST Monza Ospedale San Gerardo, Monza, Italy; ^4^ Division of Cell Matrix Biology & Regenerative Medicine, Faculty of Biology, Medicine and Health (FBMH), University of Manchester, Manchester, United Kingdom; ^5^ Università Vita-Salute San Raffaele, Milan, Italy; ^6^ IRCCS San Raffaele Hospital, Neuroimmunology Unit, Division of Neuroscience, Milan, Italy; ^7^ Pediatric Hematology/Oncology, Fondazione IRCCS Policlinico San Matteo, Pavia, Italy; ^8^ Department of Pediatrics, Fondazione IRCCS San Gerardo dei Tintori, Monza, Italy; ^9^ Department of Medicine and Surgery, University of Milano-Bicocca, Monza, Italy

**Keywords:** advanced therapy medicinal product (ATMP), potency, CAR (chimeric antigen receptor), T cell therapy, stem cell, tissue regeneration, biomarker

## Abstract

Advanced Therapy Medicinal Products (ATMPs) based on somatic cells expanded *in vitro*, with or without genetic modification, is a rapidly growing area of drug development, even more so following the marketing approval of several such products. ATMPs are produced according to Good Manufacturing Practice (GMP) in authorized laboratories. Potency assays are a fundamental aspect of the quality control of the end cell products and ideally could become useful biomarkers of efficacy *in vivo*. Here we summarize the state of the art with regard to potency assays used for the assessment of the quality of the major ATMPs used clinic settings. We also review the data available on biomarkers that may substitute more complex functional potency tests and predict the efficacy *in vivo* of these cell-based drugs.

## 1 Introduction

The use of extensively manipulated, eventually genetically modified cells, is now well established in the clinic. Among these advanced therapy medicinal products (ATMPs), perhaps the most widely used due to their clear clinical efficacy, are epithelial cells (keratinocytes) for skin or cornea repair, but others have seen important breakthroughs in the last 10 years, such as chimeric antigen receptor (CAR)-modified lymphocytes. Other cell types that have shown activity *in vivo* and reached marketing approval are mesenchymal stromal cells (MSCs), fibroblasts, chondrocytes, dendritic cells (DCs), genetically modified CD34^+^ hematopoietic stem cells and virus-specific cytotoxic lymphocytes (CTL) (1). Finally, tumor- or virus- specific T cells or NK cells, neuronal stem cells, mesoangioblasts (MABs) as well as cells derived from induced pluripotent stem cells (iPSCs) have reached the clinic and are in active development. **Table 1** lists the major cell-based ATMPs.

**Table 1:** Major cell based ATMPs that have reached the clinic or marketing authorization.

Cell type	Subtype	Uses	MA (only FDA/EMA)	Product names (year of FDA/EMA first approval)
Unmodified CTMPs (CTMP or TEP)				
Lymphocytes	CTL	Viral infections, oncology	yes	Tabcel, Anti-EBV CTL for PTLD (2022, FDA, EMA); Posoleucel, anti-viral T cells (2021, FDA)
	CIK	Oncology	no	
	Treg	GvHD, organ transplantation, type 1 diabetes	no	
	NK	Oncology	no	
MSC	BM	GvHD, organ transplantation, bone and tissue repair	no	
	CB		no	
	Adipose		yes	Alofisel, allo MSC for perianal fistules in Crohn’s disease (2018 EMA)
	Placenta		no	
DC	DCs	Vaccine effect in oncology or infectious diseases	yes	Sipuleucel-T, auto DC stimulated with prostate cancer protein (2013 EMA, withdrawn)
Epithelial cells	Keratinocytes	Repair of damaged epithelia (skin, mucosal, corneal, etc)	yes	Holoclar, auto corneal keratinocytes (2015 EMA); Gintuit, allo keratinocytes + fibroblasts in collagen (2012, FDA); Stratagraft, allo keratinocytes + fibroblasts in collagen (2021, FDA)
Bone cells	Chrondrocytes	Tissue repair in orthopedic disorders	yes	Chondroselect, auto chondrocytes, (2009, EMA, withdrawn); MACI, auto chondrocyte in collagen (2013 FDA/EMA); Spherox, auto-chondrocytes (2017, EMA)
	Osteoblasts	Tissue repair in orthopedic disorders	no	
	Fibroblasts	Tissue repair (labio-nasal wrinkles)	yes	Azficel-T, auto fibroblasts (2011 FDA)
Neuronal cells	Neural stem cells	Repair or inhibition of degeneration of neuronal tissue in Parkinson disease, multiple sclerosis, Huntington disease, Spinal cord injury etc	no	
Gene modified (GTMP)				
Stem cells	Modified CD34^+^ cells	Treatment of monoallelic genetic disorder	yes	Strimvelis, auto CD34^+^-ADA (2016, EMA); Libmeldy, auto CD34^+^-ARSA, (2020 EMA); Elivaldogene autotemcel (auto CD34^+^-ABCD1 (2021 EMA); Betibeglogene abeparvovec-xioi, auto CD34^+^ β-hemoglobin (2019, EMA)
	iPSCs	Treatment of monoallelic genetic disorder	no	
Lymphocytes	CAR-T	Cancer therapy	yes	Tisagenlecleucel, auto CART-CD19 (2018 EMA); Axicabtagene ciloleucel, auto CART-CD19 (2018, EMA); Lisocabtagene maraleucel, auto CART-CD19 (2022, FDA/EMA); Brexucabtagene autoleucel, auto CART-CD19 (2020, FDA/EMA); Idecabtagene vicleucel, CART-BCMA (2022, FDA/EMA); Ciltavabtagene autoleucel, CART-BCMA (2022, FDA)
	T-suicide	GvHD prevention in HSCT	yes	Zalmoxis, allo T-HSV-TK-ΔNGFR (2016, EMA, withdrawn)
	CAR-CIK	Cancer therapy	no	
	CAR-NK	Cancer therapy	no	

Allo, Allogeneic; auto, autologous; ADA, adenosine deaminase; ARSA, aryl sulphatase 1; ABCD1, ATP binding cassette subfamily D member; BCMA, B cell maturation antigen; BM, Bone marrow; CAR, Chimeric antigen receptor; CB, Cord blood; CIK. Cytokine induced killer cell; CTL, cytotoxic T lymphocytes; DC, Dendritic cells; EBV, Epstein Barr virus; iPSCs, induced pluripotent stem cells; MA, Marketing authorization; MSC, Mesenchymal stromal cells; GvHD, Graft versus host disease; MA, marketing authorization; PTLD, Post Transplant lymphoproliferative disorder.

ATMPs are drugs and, as such, need to be produced in specialized laboratories (i.e. Cell Factories) according to Good Manufacturing Practices (GMPs), as defined in Europe by Regulation (EC) n. 1394/2007 and subsequent EU Directives and GMP guidelines, and in the US by FDA regulations for cellular and gene therapy products (CGTs) (2, 3). These legislations aim to guarantee the safety and efficacy of the cell products for the patients. Many ATMPs used in experimental clinical trials are produced by academic Cell Factories such as ours, which have to face the challenge of complying with stringent GMP standards for novel and complex drugs, also considering the limited human and economic resources of these laboratories (2).

One important problem with ATMPs, whether produced for established use or in the context of experimental clinical trials, is the fact that these cells derive from different individuals or pools of individuals and therefore may show intrinsic phenotypic and functional variability. Potency assays are tests that are recommended to be carried out in GMP conditions on final batches of ATMPs, before their formal release for clinical use, to guarantee the effectiveness, functional quality and consistency of the cell products that will be administered to the patients (4). These potency assays may be particularly important in the context of experimental clinical trials, which aim to establish the efficacy of a specific treatment for a defined condition.

This review aims to summarize the state of the art with respect to potency assays, performed in GMP on ATMPs produced for established or experimental clinical use, and pinpoint the specific problems still present for different types of products. Whether alternative markers can be employed in lieu of more complex potency assays will be discussed, as well as possible improvements that could be applied to this field. Whether potency assays predict *in vivo* efficacy will also be reviewed. Some results obtained from our cell factory network, composed of 5 approved cell factories located in and financed by the Lombardy Region of Italy and collaborating towards the development of novel cell-based drugs for treatment of severe clinical conditions, will be summarized, to illustrate some specific observations.

## 2 Unmodified or CAR-modified cytotoxic T or NK cells or antigen-specific T-cell lines

A common type of ATMP is based on cytotoxic T lymphocytes (CTL) that are selected or induced to have specific MHC-restricted or -unrestricted cytotoxicity against specific targets. Targets can be virus-infected or neoplastic cells. MHC-restricted cytotoxicity is mediated by T cells selected for or induced *in vitro* to have anti-tumor or anti-viral activity. In some cases specificity for the target is also increased by genetic modification of T cells with defined T cells receptors (TCRs) (5). MHC-unrestricted cytotoxicity can be mediated by cytokine induced killer cells (CIKs), γδ T cells, NK, NKT or all the above cytotoxic cells, genetically modified with chimeric antigen receptors (CARs) (**Table 1**). Several of these products have been approved by FDA/EMA as marketed drugs (**Table 1**). T cells are expanded *in vitro* using IL2 and/or IL7/IL15 after different stimulation protocols. NK cells can also be expanded *in vitro* using IL2, IL15, IL12, IL18, IL21 or combinations thereof, and have been tested in clinical trials as anti-cancer agents, with limited success, in part due to limited *in vivo* persistence (6). CAR-modified NK cells are therefore an option to increase efficacy, with the advantage over T cells-based products that NK cells do not express TCRs and do not induce graft versus host disease (GvHD). Several CAR-NK products are being tested in clinical trials with some encouraging results, but none has yet received marketing approval (**Table 1**).

A common mechanism of action of T/NK cells is cytotoxicity, which is therefore commonly used as a potency assay for both cell types. Standard cytotoxicity assays include the release of measurable, cell-associated molecules, either endogenous proteins (e.g. LDH) or dyes loaded into the target cells before the test (e.g. ^51^chromium, calcein, etc) and released by the dying cells, or measuring target cell death by flow cytometry with dead/live cell dyes. In some cases surrogate markers of cytotoxic activity, expressed by the effector cells, are employed, such as the induction of degranulation markers (CD107a or granzyme B) or of inflammatory and pro-apoptotic cytokines, most commonly IFNγ, TNFα or IL2, following contact of the effector with the target cells (7). The potency tests most commonly used for T/NK cells are summarized in **Table 2**.

**Table 2:** Potency assays commonly used for cytotoxic T lymphocytes.

Cell type	Potency assays or surrogates	Common methods	Ref
CTL anti-viral	Cytotoxicity	^51^Cr release assay; IFNγ/IL2 induction by peptide loaded APC (Elispot)	(8–15)
	Specific TCR expression	Tetramer and peptide staining	(13)
	Specific Th1 cytokine secretion induced by antigen stimulation	Antigen stimulation (through peptide-or antigen-pulsed APCs) and detection of cytokine-positive T cells by flow cytometry or Elispot	(8, 10, 12–18)
CTL anti-EBV	Cytotoxicity	Cytotoxicity against EBV-LCL; Frequency of anti-EBV CTL by limiting dilution culture IFNγ production induced by target (eg Elispot)	(19, 20) (21–28)
	Specific Th1cytokine secretion induced by antigen stimulation	Antigen stimulation (through peptide-pulsed APCs or EBV-LCLs) and detection of cytokine-positive T cells by flow cytometry or Elispot	(26, 29, 30)
anti-tumor CTL or TIL	Cytotoxicity	^51^Cr release assay or flow cytometry after co-culture with tumor cells; Anti-CD3 redirected cytotoxicity of P815 cell line	(31–34)
	Specific TCR expression	Tetramer and peptide staining by flow cytometry	(35)
CIK	Cytotoxicity against autologous or allogeneic neoplastic target cells, cell lines or K562 leukemic cell line	^51^Cr, ATP, LDH or calcein release or alamar blue or flow cytometry, using dead/live markers, after 4-72 hours co-culture, at different E:T ratio	(36–42)
CAR-T	Cytotoxicity	coculture with target at different E:T ratios and assay of apoptosis by flow or ^51^Cr or dye release; IFNγ, TNFα or IL2 induction after coculture	(43–52)
	CAR expression	Percentage and/or intensity of CAR expression by flow cytometry or qRT-PCR	(43–52)
CAR-CIK	Cytotoxicity	Flow cytometry analysis with apoptosis markers after 4 hr co-culture at different E:T ratios	(31, 53)
	CAR expression	Percentage and intensity of CAR expression by flow cytometry or qRT-PCR	(31, 53)
TCR modified T	Cytotoxicity	^51^Cr release assay or IFNγ release by Elispot after co-culture	(5, 54, 55)
	TCR+ CD8 T cells	Tetramer+peptide staining by flow cytometry	(54, 55)

APC, antigen presenting cells; ATP, adenosine triphosphate; EBV-LCL, EBV immortalized lymphoblastoid cell lines; E,T, Effector,target ratio; LDH, Lactate dehydrogenase; qRT-PCR, quantitative reverse transcriptase polymerase chain reaction; TCR, T cell receptor.

Cytotoxicity assay protocols are relatively complex and quite variable between different laboratories. For example, co-culture more commonly is carried out for 4 hours in the case of dye release assays or 4-24 hours for assays based on flow cytometry. Effector target ratios used are also variable (40:1 to 1:1). Targets may be peptide-primed APCs, such as autologous PHA stimulated peripheral blood mononuclear cells (PBMCs) or DCs, or autologous tumor cells, depending on the specific T cell product, or allogeneic cell lines as surrogate targets (**Table 2**) (5, 8–14, 19–27, 29, 31–33, 36–55). Another, non-specific cytotoxicity assay indicating the presence of functional cytotoxic T cells within the product, irrespective of antigen specificity, is the anti-CD3 redirected lysis of the NK-resistant, FcγR^+^ P815 cell line (34, 56).

Surrogate markers of cytotoxicity include, for expanded antigen-specific T cells, tetramer staining and flow cytometry which measures the frequency of cells expressing TCR against a specific peptide presented on an appropriate MHC (13, 31, 35). For polyclonal T cells it does not therefore identify all the effector cells that may mediate cytotoxicity against a specific target. For gene modified (TCR or CAR) cells, the TCR and CAR can be quantified by flow cytometry or quantitative PCR (pRT-PCR) and are therefore more accurate surrogate markers of potency, as they should detect all target-specific T cells within the product (**Table 2**) (31, 43–53). Regarding virus specific ATMPs, another surrogate marker of potency is the detection by flow cytometry or by Elispot of Th1 cytokines, such as IFNγ, secreted by the T cells upon stimulation with the specific antigen. The Th1 cytokine-secreting function is considered a marker of cell activation and specific activity, and, although it does not directly reflect the presence of cytotoxic T cells, it is likely to be associated to immune protective activities, such as lytic function (8, 12, 15–18, 30) (**Table 2**).

**Figure 1:**
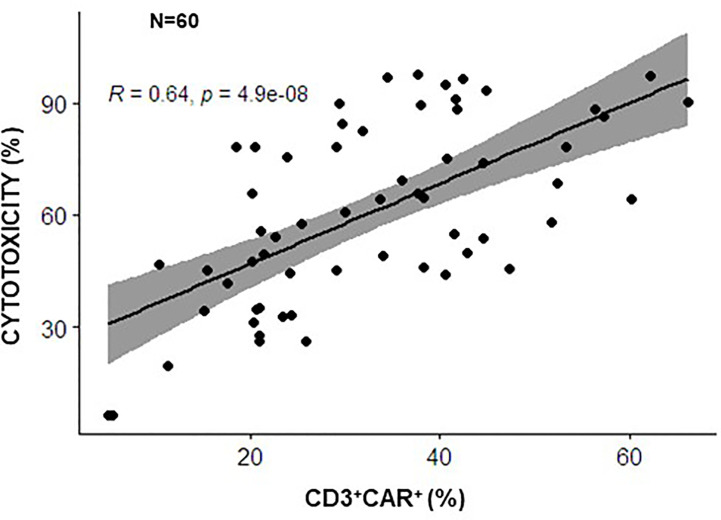
Correlation between CAR expression and the cytotoxic potential of CARCIK-CD19 cells. CARCIK-CD19 cells expanded for clinical use of validation purposes, using the same GMP standard operating procedure, in 2 different Cell Factories (Center of Cellular Therapy “G. Lanzani, Bergamo and Laboratory of Cell and Gene Therapy Stefano Verri, Monza, Italy), in the context of common clinical trials (NCT03389035 and NCT05252403), were tested for percentage of CAR-CD19 expression and cytotoxic activity against the REH leukemic cell line, using the same assays conditions (N=60). Percentage CAR expression correlated significantly with percentage cytotoxicity (potency) (The Pearson correlation coefficient R=0,64, p<0.0001).

Whether surrogate markers correlate with functional potency *in vitro* is an important issue in the context of clinical trials and with the view of defining predictive markers of response *in vivo*. Regarding gene-modified T cells, whether the quantitative assay of CAR expression reflects the functional potency *in vitro* of CAR-CIK cells has been investigated by our groups. The percentages CAR-CD19 positive cells was demonstrated to correlate significantly with cytotoxicity *in vitro*, performed against a standard CD19^+^ B-acute lymphoblastic leukemia (ALL) cell line, in the context of our two clinical studies using 60 batches of CIK cells modified with a CAR anti-CD19 (NCT03389035 and NCT05252403 and product validation data for these trials) (**Figure 1**). These data suggest that quantitative analysis of CAR expression can indeed be used as a rapid and surrogate potency assay for GMP release, instead of more laborious cytotoxicity assays.

Given the fact that functional potency assays are quite laborious and may show a relatively high level of variability, as detailed above, they are sometimes employed only in the development and validation phase of ATMPs and not used as a routine GMP release test during clinical experimentation. In this latter context, they may be used “for information only” (FIO), for example to evaluate retrospectively whether *in vitro* cytotoxicity correlates with the clinical response or to perform statistical analyses of production efficiency. Indeed, presently, there are no standardized and universally recognized potency methods or parameters for GMP-compliant batch release of T- or NK- cell products. Specifications may also vary between laboratories and depend upon the results of analyses made during development. In contrast, surrogate tests of the cells overall fitness are universally applied. For example, T cell overall fitness is measured by testing the cell product viability, as well as the immunophenotype that defines the ATMP product identity, detecting as a minimum the CD3, CD4 and CD8; defined TCR or CAR expression are also measured in case of genetically modified T cells.

More extensive characterization of the T cell based ATMPs, including the quantification of naive-memory subsets, the expression of co-stimulatory molecules or checkpoint inhibitors and other makers that may indirectly correlate with potency are usually performed only as ancillary tests. In particular these assays are used to gain knowledge of the product characteristics and to allow retrospective correlative studies that may explain the clinical response of patients (45, 47, 48, 57, 58). There are relatively few data reporting whether potency assays or surrogate markers of functionality do correlate or not with the efficacy of T- or NK-cell based ATMPs *in vivo* in the clinic. The data available on CAR-T cells, mostly directed against CD19 and used for the treatment of acute lymphoblastic leukemia (ALL) and B-non Hodgkin’s lymphoma (B-NHL), are however informative in this regard. The published results show that *in vitro* cytotoxicity, CAR expression, CAR-T dosage or CAR-T CD4/CD8 ratio in the product do not usually correlate significantly with the clinical response (59, 60). Rather CAR-T cell expansion *in vivo* following cell infusion, and the persistence of a less exhausted phenotype of the cells recovered at different time points after infusion, predicted response *in vivo* (60, 61). Also patients and tumor characteristics such as tumor burden, state and frequency of circulating T cells upon infusion, tumour microenvironment parameters, including expression of checkpoint inhibitors or presence of immune suppressive cells (62–64), have been observed to be predictive factors of the clinical response (19, 57, 58, 65, 66).

However, in the last 5 years, quite extensive analyses of the characteristics of the infused CAR-T cell products have been conducted in the context of clinical trials, probing more complex phenotypes, in some cases using single cell RNA sequencing (scRNAseq) or epigenetic analyses, in order to identify characteristics of the cell products that may predict *in vivo* cell expansion, persistence and clinical response. These investigations have demonstrated that an early memory and less exhausted phenotype of the infused cell product (60, 67–69), a high frequency of CD8^+^CD27^+^PD1^-^ cells showing an upregulated STAT3/IL6 pathway (60), polyfunctionality (70, 71), shorter effector-target contact and higher out-of-contact migratory capacity (71), IFNγ secretion (70), a Th2 profile following activation *in vitro* (67), as well as a specific epigenetic pattern of DNA methylation (72), are all biomarkers that may predict in part the persistence of the infused cells and/or the clinical response to CAR-T therapy. Homing receptors such as CCR7 and CXCR3 have also been suggested to correlate with clinical response but results are still controversial (60, 67, 70). Indeed despite the extensive characterization of chemokine receptors, such as CXCR1, CXCR4, XCL, CCL3/4 and 5 for the recruitment of cells to different tissues *in vivo* (73), there is still a lack of clear understanding of their effective role in the context of passively administered T/NK cells (unmodified or gene modified) for their effective intratumoral recruitment. Altogether, the present data on product characteristics confirm that batch variability does exist and that parameters other than CAR expression and *in vitro* cytotoxicity need to be applied to better characterize the efficacy of this class of ATMPs. Furthermore the more precise characterization of an effective CAR-T product will allow to define improved expansion conditions to more reproducibly generate less exhausted and more effective cell products (69, 74). Indeed the quality of the starting cell material used to expand CAR-T cells, as well as specific CAR and expansion conditions, may influence the quality and efficacy of the final CAR-T cells (60, 74, 75). Clearly, further work and larger studies will be required to confirm all these data and define appropriate GMP compliant markers of CAR-T potency that may be applied to clinical grade CAR-T cell products, to improve their reproducibility and quality. Similar work is warranted for MHC-restricted T cells and NK/NKT cells used in clinical trials. New technologies and *in vivo* models may also be useful to better understand the dynamics of T cell function and define predictive parameters of efficacy *in vivo* (76–78).

NK and T cells have also been generated *in vitro* from iPSCs for immunotherapeutic purposes (79, 80). The expansion potential of iPSCs eliminates the need for multiple donors, increasing cell product reproducibility, and epigenetic rejuvenation during iPSC should produce biologically younger cells (81). These may therefore also become “off-the-shelf” products. Nonetheless, despite impressive progress to obtain meaningful number of cells using simplified steps applicable to GMP conditions (82), very few clinical reports have yet been published so far with these ATMPs (59, 60, 83, 84). Furthermore, the frequent chromosomal alterations as well as the teratomas observed in mouse experiments may represent a severe limitation to current clinical translation (79, 80), suggesting the necessity of adding suicide genes for safety issues.

iPSCs can be established from T-cell clones and re-differentiated into functional T cells. CD8^+^ T lymphocytes derived from iPSC with specificity for tumor antigens, such as MART-1, or viruses, such as HIV-1, have been generated using this technique (85). These rejuvenated CD8^+^ cells have shown IFN-ɣ production and cytotoxicity against the relevant targets, improved proliferation capacity and a less differentiated profile associated with prolonged persistency with respect with unmodified T cells (85–89). Potency assays for iPSCs generated T or NK cells are the same as those for standard cells.

Suicide systems are available for both unmodified or CAR-T cells and include the introduction of proteins (CD20, HSV-TK) that can be targeted by drugs (rituximab, gancyclovir) or inducible pro-apoptotic protein such as iCasp9 (90). Clearly potency assays also need to verify gene copy number, level of cell transduction, the effective expression and/or function of the suicide gene, depending on the system used. Few such suicide systems for ATMPs have yet reached the clinic (91, 92).

## 3 Dendritic cells

Different subsets of DCs are present in man, such as conventional DCs, plasmacytoid DCs and Langheran cells, each with specific phenotypes, localization and functions. Conventional DCs are very efficient antigen presenting cells (APCs) that can be relatively easily generated *in vitro* by culturing monocytes (usually derived from peripheral blood), in presence of appropriate cytokines, most often GM-CSF and IL4 for the differentiation phase to generate immature DCs (iDCs), followed by a 24-48 hour culture in presence of TNFα, IL1β, IL6 and Prostaglandin E2 (PGE2), to generate mature DCs (mDCs) (93, 94). These *in vitro* generated cells are CD1a/CD80/CD83/CD86/CD40/CD209^+^, HLA-DR^+^, express MHC-class I cells and are called monocyte-derived DCs. Mature DCs process and present peptides from intracellularly processed proteins on MHC class I/II complexes, in cooperation with the costimulatory molecules (CD80/CD86/CD40) and pro-inflammatory cytokines (IL12), and thus prime T cells. These cells, once loaded with antigens, peptides or cells (for example viral antigens or tumor cells), can induce Th1 responses as well as activation and proliferation of naive or memory CD8^+^ cells to induce CTLs directed against the presented antigens. Antigen processing, co-stimulatory signals, in particular CD40, CD80/CD86 and cytokines production (mostly IL12) are essential for effective T cell stimulation and polarization toward an effective immune function.

Unmodified DCs have been extensively used in clinical trials as vaccines with the scope of inducing or boosting T cells response against tumors or infectious agents (95, 96). However, regulatory or tolerogenic DCs can be induced by a tolerogenic tumor environment which may negatively influence the efficacy of autologous mDCs, including DCs generated *ex vivo* in GMP (95, 97). Due to the still relatively limited success of unmodified DC-based vaccines (98), more recently, gene-modified DCs are being generated with the hope of enhancing their efficacy *in vivo*. Genetic modifications include the introduction by viral infection or transfection of the desired target antigens (viral or tumor antigens), with or without a co-stimulatory molecule, or of the cDNAs encoding the GM-CSF/IL4 cytokines that will permit the efficient generation of “self-generated” iDCs from immature precursors (SmartDCs) (99–103).

Potency assays for DCs generally measure allogeneic mixed lymphocyte reaction (MLR) *in vitro* and assay conditions and detection methods vary quite significantly between laboratories. Furthermore, such assays, which are based on proliferation measured over several days and employ different donors, show intrinsic variability. Specifications also vary between laboratories and are based on the validation data (31, 93, 94, 104, 105) (**Table 3**). Thus, no standardized assays are defined for DCs. Potency assays for genetically modified DCs are similar to those used in the context of unmodified DCs, since the desired function of these ATMPs is the same, and the same caveats apply, i.e. variability and lack of accuracy in the assays; they may additionally include quantitative evaluation of the introduced genes by quantitative PCR (99, 100, 102). Potency assays may in some cases be used as a GMP release assay but are more likely employed in the validation phase and/or used for information only in the context of clinical trials. They may be performed before cryopreservation or on cryopreserved cells (31, 93, 94, 104, 105). Immunophenotyping to define the identity of the clinical grade DC products is therefore often used as surrogate for potency assays (positivity for CD11c, CD80/83/86, HLA-DR, CD209 and negativity for CD14, CD3 CD19), but the set of markers used and the specifications somewhat vary between laboratories (31, 93, 94, 104, 105).

**Table 3:** Most common potency assays for DCs.

Cell type	Potency assays or surrogates	Common methods	Ref
DCs or genetically modified DCs	Target specific T cell proliferation; One-way MLR; T cell mediated cytotoxicity after coculture with DCs; IFNγ stimulation; Phagocytic capacity	Co-culture at different E:T ratios and T cells proliferation measured by flow cytometry, ^3^Thymidine or tetrazolium dye assays in presence of peptide or target cells loaded DCs; IFNγ production by co-cultured T cells measured by Elispot; Phagocytosis of zymosan particles	(31, 93, 94, 104, 105)

MLR, Mixed lymphocyte reaction; E:T, Effector:target ratio.

Whether the potency of a DC vaccine may be predictive of *in vivo* efficacy is not clear. Several groups have shown increased T cell responses and cytokines, or decreased Tregs post DC-based vaccination, which may or may not correlate with the clinical response (98, 106–109). There is also some indication that the presence *in vivo* of lymphocytes that can be activated by pulsed DCs to become cytotoxic against the autologous tumor correlates with the clinical outcome of ALL patients, but these patients were not treated with a DC vaccine (110). Other groups have shown a correlation between *in vitro* potency or surrogate markers thereof and clinical response (101, 111). These data need to be confirmed on larger cohorts of patients.

To summarize, potency assays are performed for DCs intended in a vaccine mode to induce T cell mediated immunity against tumor cells or virus infections. These are performed in the validation phase and in some cases also for release of the GMP grade ATMPs. Assays are quite variable between laboratories and are not standardized. Furthermore, few data are yet available to demonstrate that potency *in vitro* predicts efficacy *in vivo*. More extensive characterization of the cell products, similar to what has been done for T cell-based products, is warranted to define better biomarkers predictive of efficacy.

## 4 Mesenchymal stromal cells

MSCs have been extensively expanded *in vitro* for clinical use, due to the ready availability of the tissues from which they can be derived: mostly bone marrow (BM), adipose tissue (AT) and newer sources such umbilical cord (UC), cord blood (CB), placenta (PL), amniotic fluid or amniotic membrane (112–115). In addition, MSCs show multiple functions *in vitro* and *in vivo*. In particular, they display immunosuppressive properties acting on different cell types, as well as a capacity to differentiate to adipose or bone tissue, making these cells potential drugs in many different clinical contexts. MSCs, expanded *in vitro* according to a variety of different protocols and starting from different tissues have therefore been extensively used in the clinic to treat a variety of conditions. Three MSC products have been approved worldwide: Alofisel, an allogeneic adipose derived product from Takeda and TiGenix approved by EMA to treat enterocutaneous fistulae (**Table 1**). In addition, two MSC products have been approved in Korea: Cartistem, based on human CB derived MSCs for the treatment of knee cartilage defects, as a result of degenerative osteoarthritis or repeated trauma and Cupistem, which is an autologous adipose tissue derived MSC product for Crohn’s Fistula. None have yet been approved by FDA (112, 116–120). Unfortunately, most of the clinical applications of MSCs have not yet produced very significant and/or reproducible clinical results (113, 121).

Potency assays performed on MSCs are different according to the expected *in vivo* function of these ATMPs, i.e. whether their pharmacological use is based on their immunosuppressive (122–124) or tissue repair properties (31, 124–128). However, MSCs’ mechanism of action *in vivo* may involve the effect of many effector pathways with synergistic and overlapping functionalities. For example, dominant MSC functionalities in immune modulation may overlap with those relevant in tissue injury response, with some unique components for each. Considering this, it has been difficult to define and identify a cellular universal reference standard that would contemporaneously display all of the potential functions of MSCs identified to date (129–131).

**Table 4** summarizes the potency tests that are most commonly applied to MSCs in different clinical contexts. Some are routinely used for release assays, others have been described and employed only in the development and characterization phase of the cell product and are not performed on clinical batches for GMP release, since the development of an appropriate potency assay is challenging (132). Indeed induction of IDO, PGE2, adhesion molecules or the transcription factor TWIST as well as others by inflammatory mediators have been demonstrated for MSCs, but their detection relies on complex assays so far described only in the preclinical development phase (133). Furthermore, the methodologies and findings may vary markedly between different laboratories (134). Other tests are qualitative more than quantitative (e.g. the differentiation potential to different cell types; **Table 4**) (31, 124, 126–128). One approach to reduce the observed batch to batch variability when testing for functional assays of MSCs is to generate batches from pooled donors. This approach has been suggested to result in more reproducible products (119). These data will need to be confirmed in larger studies.

**Table 4:** Most commonly used potency assays to functionally qualify MSCs.

Intended clinical use	Potency assays	Common methods	Ref
Immunosuppression, e.g.in the context of autoimmune diseases, organ transplantation, including GvHD treatment or prevention post-HSCT	Inhibition of T cell proliferation induced by PHA	Measurement of T cell proliferation by fluorescent (CFSE, BrDU), tetrazolium dyes or bioluminescent reagents etc, after co-culture with PBMCs	(122–124)
	Inhibition of Th1 T cells activation	Overnight coculture of MSC and PBMC and stimulation of Th1 cells. Inhibition of IFNγ production by CD4^+^ Th1 or CD3^+^ T cells by flow cytometry	
	Inhibition of release of cytokines (TNFα, IFNγ)	ELISA measurement of cytokines after coculture and stimulation with PHA	
	Release of immunomodulatory cytokines (IL6, IL8)	ELISA of MSC conditioned medium	
	Molecular genetic and secretome analysis	Supernatants from 4-days coculture of MSC and PBMC analyzed by multiplex luminex assays for cytokines, chemokines, and growth factors. Evaluation of RNA expression by Quantitative PCR of selected gene products expressed by MSCs upon interaction with either PBMCs or IFNγ.	(129)
Tissue repair (mostly bone and cartilage)	Adhesion to solid supports	Adhesion to plastics or biomaterials	(31, 124, 126–128)
	Proliferation *in vitro*	Cell count after *in vitro* growth for varying time	
	Differentiation *in vitro* to adipose, osteoblasts or chondrocytes	*In vitro* differentiation for 11-21 days in presence of appropriate media and supplements followed by staining with appropriate reagents to identify differentiated cell populations	
	Angiogenesis assay	Effect of MSC conditioned medium on angiogenic assays using HUVEC	
	Release of VEGF and HGF	ELISA of MSC conditioned medium	

ELISA, Enzyme-linked immunosorbent assay; HUVEC, human umbilical vein endothelial cells; MLR, Mixed lymphocyte reaction; PHA, Phytohemagglutinin; PBMC, Peripheral blood mononuclear cells.

With regard to the tissue regenerating function of MSCs, potency assays are usually performed only to demonstrate the capacity of the expanded MSCs to differentiate into the expected cell types, depending on the specific clinical application (e.g. bone or cartilage repair) (31, 124, 126–128, 135).

Whether the GMP release assays correlate with the clinical response to the MSC infusion is still unclear. Indeed little correlation has been observed between inhibition of proliferation *in vitro* and immunosuppressive function *in vivo*, or between differentiation potential in the test tube and tissue repair (summarized in (4, 133)). Similarly, reliable *in vivo* markers of disease and response to MSCs are still lacking (133, 136). With regard to the immune suppressing function of MSCs, this is in part due to a still incomplete understanding of the MSCs mechanisms of action *in vivo* in different disease contexts. Chinnadurai and colleagues have tested an *in vitro* assay matrix approach combining molecular, genetic and secretome analysis, elements of which could be deployed to define MSC immune modulatory potency (129). Indeed MSCs may need to respond to *in vivo* clues to become activated and unleash their full potential (113, 125). Moreover, recent data suggest that apoptosis of infused MSCs and their phagocytosis by macrophages, called efferocytosis, may trigger immunosuppressive cascades of events and may be more important than the direct immunosuppression by the MSCs themselves (133, 137, 138). Indeed the apoptosis of MSCs induced by the PBMCs of patients has been suggested to be a better predictor of *in vivo* MSC activity (138, 139). Finally, evidence indicates that another mechanism of MSC function is *via* the release of extracellular vesicles by these cells (EVs, see below) (139).

MSC products are heterogeneous cell populations which are known to gradually lose their multilineage and proliferation potential during culture in adherent monolayers (140–142). Indeed the ISCT has recommended the term mesenchymal stromal cells rather than mesenchymal stem cells, due to lack of evidence of real stemness of the cells, i.e. the capacity to self-replicate and differentiate into different cell progeny (143). In addition, no marker of stemness or of differentiation is available to define the MSC precursors present in culture and their capacity to proliferate and differentiate to the desired tissue. Such lack of marker makes the further development of MSC-based therapeutics more difficult at present (136, 144, 145).

In this context, the lack of clear knowledge of relevant potency assays to define MSCs activity *in vivo*, as well as their intrinsic variability and complexity, has led many groups to propose as release assays the sole immunophenotypic and viability analyses, i.e. the definition of a minimum phenotype and vitality of MSCs, which are part of the ISCT recommendations for this ATMP (31, 146). The consensus ISCT criteria for MSC identification is positivity (≥ 95%) for CD105, CD73 and CD90 and negativity (≤ 2%) for CD45, CD34, CD14 or CD11b, CD70a or CD19, and HLA-DR. Some groups have used less stringent immunophenotypic criteria for GMP purposes and do not apply all recommended markers in this context (e.g. ≥80%, ≥85%, ≥90%, positivity for CD73, CD90 and CD105) (31, 126).

The identification of markers of stemness and/or lineage differentiation potential, similar to what has been identified in epithelial stem cells (see below) should continue to be pursued also for MSC products.

EVs have been produced from MSCs for therapeutic use and in GMP conditions. given the reported functional activity of these products (139, 147). EVs are purified and used in lieu of live cells and are not therefore strictly ATMPs, but ATMP-derived, and are still in search of classification (148). They are however presently extensively tested in clinical studies across different therapeutic areas (149–151). Their qualification and potency evaluation should follow the recently published guidelines, that include expression of at least 3 markers as well as physical characterization (152). Indeed MSC derived EVs express markers such as CD9, CD63, CD81, TSG101 and do not express calnexin and cytochrome C (147, 151). Physical characterization of particle size and concentration should be evaluated by two different but complementary techniques. These EVs are thought to act *via* paracrine intercellular communication effects, inducing tissue repair and immunomodulation by different mechanisms. Functional potency assays for EVs show similar limitations to that described for MSCs and their relevance to the *in vivo* efficacy is still a little understood (139).

## 5 Epithelial stem cells

Autologous epithelial cells expanded *in vitro* from different tissues and organs have been used extensively for the treatment of severe burns and were the first success of extensively manipulated cell-based drugs (136, 153–156). These autologous epithelial stem cells are marketed for tissue repair as Holoclar, whereas allogeneic epithelial cells are marketed as Gintuit and Stratagraft (**Table 1**).

Successful engraftment of keratinocytes in culture depends upon the presence and amplification of epithelial stem cells capable of forming holoclones (156). *In vivo*, stem cells are usually slowly cycling and can give rise, after isolation, to distinct epithelial lineages characteristic of the different tissues from which they were derived, even after *in vitro* culture (157). Stem cells for corneal epithelium reside in the limbus whereas stem cells for conjunctival epithelium reside in the bulbar and forniceal conjunctiva and generate both conjunctival keratinocytes and goblet cells, both of which are important for the integrity of the ocular surface (158). Stem cells for skin epithelium are distributed in the basal layer of interfollicular epidermis and various niches of the hair follicles. Stem cells are also found in mucosal epithelia (136, 158–160).

Epithelial stem cells can be expanded *in vitro* according to a variety of culture conditions and show a high proliferative capacity (up to 80-160 cell divisions *in vitro*). Final products are characterized for research purposes by cell number, clonogenic assays (to measure the frequency of holoclones as well as of more differentiated meroclones and paraclones) and analyses of the proliferative and differentiative capacity of these clones (136). Stem cells (holoclone forming cells) also express high levels of stem cell markers, most specifically ΔNp63α, but also other less specific markers of stemness such as vimentin, ABCB5 and keratins CK14 and CK15 (see below) (161–163). Differentiation *in vitro* to different epithelial types can be defined by staining for other keratins specific of different tissues (158). More recent technology includes the generation and transplantation of cellularized scaffold products such as StrataGraft or Gintuit (sheets of allogeneic human keratinocytes and fibroblasts in collagen), the investigation of pluripotent embryonic (hPSCs) or induced pluripotent stem cells (iPSCs) to generate epithelial cells *in vitro* and on improved culture conditions, substrates and co-cultures to expand epithelial sheets containing stem cells able to differentiate into different epithelia (155, 161, 164–167). Allogeneic material, generally containing also fibroblasts, is used for temporary skin replacement until autologous cells can be generated and administered (168). Finally other developments are the genetic modification of epithelial stem cells *in vitro* to correct monosomic genetic diseases affecting the skin (136).

A fundamental aspect of epithelial stem cell use for tissue regenerative purposes in the clinic is to define the content and frequency of stem cells (holoclones) within the cell graft, i.e. a measurement of potency of the product (136, 156). Although colony assays can be used to functionally characterize the cell-based drug, these are long and laborious and are not always suitable for GMP release purposes. They are therefore usually performed in the product development/validation phase or for information only during clinical trials. A number of phenotypic markers have therefore been identified to try and define epithelial stem cells as well as their differentiated progeny. The p63 transcription factor is a regulator of epithelial proliferation and has been shown to be the best marker of stemness in epithelial cell cultures. Indeed the ΔNp63α isoform is a marker of corneal, epidermal, oral and conjunctival epithelial stem cells, i.e. holoclones (136, 163, 169, 170). Furthermore this marker is the only marker so far that correlates with the clinical response to limbal stem cells (171, 172). It is therefore routinely used to measure the number of stem cells in epithelial cell products for clinical use. Other, far less clear-cut, parameters for stem cells are small cell size, positivity for ABCG2, cytokeratins CK14 and CK15 and/or integrin α6 and negativity for differentiated epithelial cells (CK1, CK10, CK3, CK12 and CK19) (163, 173–177). Some groups have also attempted to standardize these assays, adding reference standards, and define specifications values (170). Recent evidence suggests that proliferative capacity *in vitro*, as well as the presence of stem cells strongly expressing ΔNp63α (p63^bright^), may both be important factors to predict epithelium regeneration *in vivo*. Therefore such parameters should be investigated in clinical studies to further improve the characterization of the cell products and identify factors that determine successful engraftment (136, 163, 178). Finally, additional work is being conducted to further improve safe and more effective culture methods for generation of epithelial stem cells for clinical use and potency assays are important in this context as well (136, 178).

Also in the case of genetically modified epithelial stem cells to correct monosomic diseases, the evidence is compelling that long-term reconstitution of corrected keratinocytes depends upon the presence of holoclones (179). In the case of genetically modified cells, clonal analyses of potency are usually performed on the product in parallel with product administration (in fresh). The correct expression of the introduced gene is additionally performed by qRT-PCR or protein analyses.

We conclude that it would be useful to standardize the detection assay for ΔNp63α and routinely add such potency test marker for all epithelial products used in clinical trials, in order to allow correlative studies to be made and establish whether this marker can predict the engraftment and duration of the graft (136, 157).

## 6 Neuronal stem cells

In the last 30 years, the generation *in vitro* of large numbers of neuronal stem cells (NSCs) from fetal brain tissue has been developed in order to treat few degenerative or post-trauma neural diseases (180, 181). Ethical and regulatory issues, as well as difficulties in reproducibly expanding standardized cell products have delayed the clinical application of these products, but in recent years Phase I or II studies have been performed with non-genetically modified, *in vitro* expanded, GMP-grade NSCs in different clinical contexts, in particular Parkinson disease (PD), amyotrophic lateral sclerosis (ALS), multiple sclerosis (MS) and spinal cord injury (SCI) (182–185). Human NSCs are generated by extensive culture expansion in presence of EGF and bFGF, which lasts from few weeks to several months (186). NSCs can differentiate into neural and glial cells (astrocytes and oligodendrocytes) *in vivo*, and can produce neurotrophic factors, cytokines and extracellular vesicles (EVs), promoting neuronal tissue repair, immunomodulation, or neuroprotection (187). Defining potency assays for such products is therefore particularly difficult, given the multiple and not yet fully defined mechanisms of action of these cells, which may vary in different disease contexts and in the presence of multiple factors in the host environment. Thus, similarly to MSCs, potency assays *in vitro* and *in vivo* relevant to the intended target disease should be performed (Potency Tests for Cellular and Gene Therapy Products | FDA. https://www.fda.gov/regulatory-information/search-fda-guidance-documents/potency-tests-cellular-and-gene-therapy-products).

This is obviously a difficult task, specifically for assays performed *in vivo* in small animals, due to its labor-intensive nature. The *in vivo* models are often preferred over *in vitro* systems due to their highest level of biological complexity, being the most relevant to the clinical settings. Potency assays *in vivo* differ according to disease model, but usually include verification of infiltration of human NSCs or graft survival into different brain regions of treated immunodeficient mice or rat models, 3-6 months after NSC transplantation (186, 188). As clinical outcomes, partial reversal of neurodegeneration or neuronal repair are investigated using various functional tests at different time points following NSC administration. These tests are model-specific, thus difficult to standardize across different laboratories.

On the other hand, tissue- or cell-based potency assays help to reduce the use of animal models according to the 3R principle (replacement, reduction and refinement) and can directly evaluate the functionality of the cellular product. *In vitro* potency assays include growth rate and capacity to form embryoid bodies or colonies at low density (186); however culture conditions and growth rate evaluations can substantially vary between laboratories. Additional *in vitro* biological potency assays may include the evaluation of surface markers expression, release of soluble mediators and changes in gene transcription. Current evidence suggests that performing potency assays with clinical grade NSC cell lines, in parallel with reference standard cell lines, should be mandatory before performing clinical trials, as nicely described by Anderson and colleagues (188). Furthermore, transparency and full functional description of the biological functions tested for the cell products used in clinical trials should be included in the report of clinical efficacy or lack thereof, in order to allow a correct interpretation of positive and negative results and further advance the field (188). Nonetheless, progress has been made in the last 10 years to better define the origin of the NSC starting material used for expansion, and more standardized culture protocols have been proposed by some groups (186). Importantly, sufficient NSC numbers can be obtained from a single donor, which are then expanded to create the master cell bank and cryopreserved, allowing to perform larger clinical trials and to treat hundreds of patients with rigorously quality-controlled cell products. The number of cells generated in large batches for each expansion (each cell line) is sufficient to perform more extensive *in vitro* and *in vivo* potency assays before clinical use (183, 186). Nonetheless, cellular products even obtained from a single donor typically show a large degree of lot-to-lot variability, due in part to inherent variability of the starting material, donor genetic factors, epigenetic changes and genetic polymorphisms. Therefore, human iPSCs differentiated *in vitro* to neural precursor cells (NPCs) can be employed as an alternative to NSCs of fetal brain origin (180, 189).

In the past years, the number of iPSC-based clinical trials have increased dramatically (190), including the first case report of iPSC-derived dopaminergic precursors transplantation in Parkinson’s disease patients (191). The majority of clinical trials using iPSC-derived products are being conducted in an allogeneic setting, since the generation of iPSC under GMP conditions (required for clinical use) for a single patient is time-consuming, laborious and not cost-effective, despite the fact that initially iPSCs were intended to be used in an autologous setting. Therefore, iPSC banks with common HLA haplotypes have been established in several countries, to advance the development of cell therapies using iPSC-based products (192, 193).

Before clinical use, iPSCs and their products are rigorously controlled for the absence of adventitious viruses and mycoplasma. Chromosome analysis (karyotyping), DNA fingerprinting, and whole genome sequencing performed to control for absence of cancer-related and neurodegenerative-associated mutations. Detailed genetic integrity analyses of iPSCs are described by Popp and colleagues (194). Furthermore, the final iPSC-derived products are quality controlled for absence of undifferentiated iPSCs, accurately characterized *in vitro* by immunocytochemistry (NESTIN, SOX2 neural progenitor markers expression) and RT-PCR (absence of OCT4, SSEA4 expression) and functionally assessed with electrophysiology analyses. Human NPCs can be generated with a two-step approach from iPSCs, however the presence of even few undifferentiated iPSCs in the final cell product could lead to tumor formation upon transplantation. To address this issue, genetically modified NSCs are being proposed for clinical use, including more stable NSC cell lines (136, 189). Genetically engineered NSC show an enhanced capacity to produce the specific therapeutic molecules (e.g. LINGO-1-Fc, IL10, NT-3 or TGFβ1) to counteract neuroinflammation and promote tissue repair (195). The direct differentiation of human fibroblasts towards neural progenitors for clinical use, *via* viral delivery of transcription factors to generate iPSCs, have intrinsic potential safety risks. For this reason, novel methods of direct differentiation *via* non-genetic chemical modulation or by transgene-free delivery of transcription factors using mRNA or proteins have been developed (196, 197). The induced NSCs (iNSCs) obtained have showed efficacy in several preclinical studies, including stroke, Parkinson’s, and Alzheimer’s disease, multiple sclerosis and brain cancer, however no clinical data of these cells have been reported up to date (198).

The therapeutic potential of neural stem cells is undoubtedly encouraging but further development of GMP-grade standardized protocols for NSC differentiation and characterization are urgent. Better understanding of NSC mechanisms of action will allow to develop more efficient potency assays to ensure cellular product safety and efficacy in the clinical phases.

## 7 Mesoangioblasts

A successful therapy for patients suffering from different muscular dystrophies is still missing. In the case of cell therapy, initial studies attempted to inject satellite cells (muscle stem cells)-derived myogenic progenitors from a parent into a single muscle of patients affected by Duchenne Muscular Dystrophy (DMD). However, the limited survival and migration of the injected cells into the tissues led to disappointing results. More recently however, injection of autologous myogenic progenitors (isolated from non-affected muscles) in the pharyngeal muscles of patients affected by Facio-Scapulo Muscular Dystrophy (FSMD) resulted in a significant amelioration of swallowing, the main problem of these patients (199). Other muscle stem/progenitor cells were subsequently isolated and tested mainly intra-muscularly in dystrophic, immune deficient rodents (200). Mesoangioblasts (MABs) are a subset of muscle pericytes (201) that can be expanded extensively in culture and maintain the ability to differentiate into skeletal and smooth muscle cells. Of relevance, they express some of the proteins that leukocytes use to bind and cross endothelium in the presence of inflammation, which suggested the possibility of a systemic, intra-arterial delivery, not possible with other myogenic cells. Preclinical work in dystrophic mice (202) and dogs (203) led to a first in-man trial, that proved safe but of very limited efficacy (204, 205).

In particular a current promising approach investigates the clinical use of muscle pericyte-derived MABs (206). Indeed, one of us (GC) has developed culture conditions to generate sufficient number of genetically modified autologous MABs for a clinical trial in patients with DMD (Acronym: DMD06-Mab). The cells are genetically modified with a lentiviral vector expressing the U7 small nuclear RNA, engineered to skip exon 51 of the dystrophin gene. The MABs can be expanded *in vitro* up to approximately 20 population doublings (PD), maintain an euploid karyotype up to senescence and do not form tumors when injected subcutaneously into nude or SCID mice (201).

**Figure 2:**
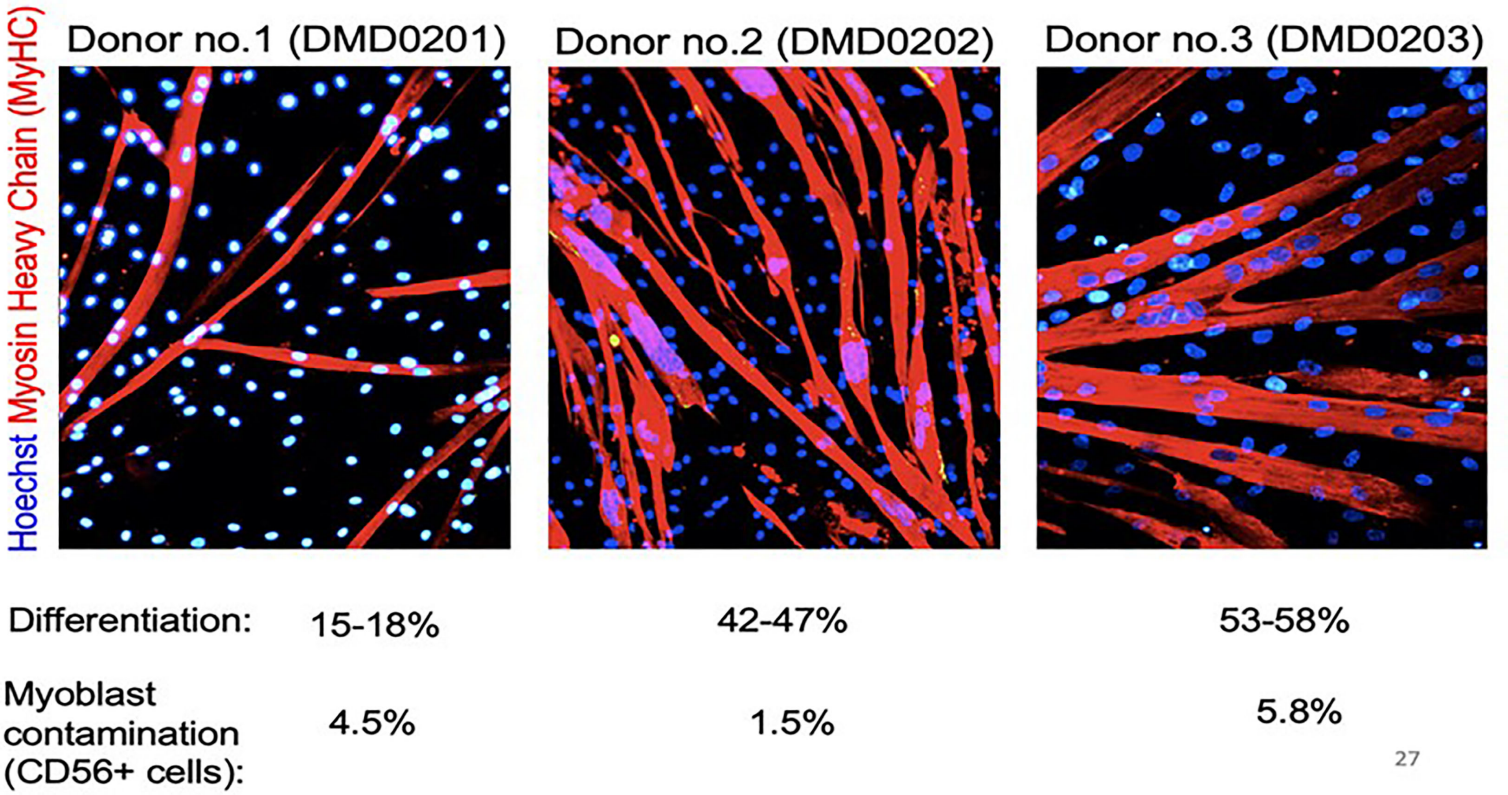
Donor MABs form myotubes *in vitro*. Spontaneous differentiation of donor MABs obtained from the medicinal product before infusion. 2x10^5^ MABs were plated on low-growth factor matrigel coated 3.5 cm Petri dish, in proliferation medium (Megacell). After an O/N incubation at 37°C, 5% CO_2_, proliferation medium was replaced by differentiation medium (DMEM supplemented with 2% horse serum), and differentiation extended for 10 days. Cells were stained with anti-myosin heavy chain antibody and Dapi. Reprinted from (204).

MABs express several stem cell surface markers but these are not specific for MABs (200). Adult MABs express PW1/Peg3 which is one of the drivers of muscle differentiation. Nonetheless, potency assays for gene-modified MABs rely presently on rather complex and long functional assays carried out on an aliquot of ATMP product to be administered to the patient (206). Potency assays include *in vitro* differentiation for 5-10 days in appropriate media and measurement of dystrophin production (207, 208). Dystrophin expression can be tested at the level of the RNA or protein level. The first is more quantitative but may detect abnormal transcripts that will not be translated (depending on the specific mutation), while western blot detects the protein, but is semi-quantitative. The differentiation potency can also be revealed by immunofluorescence with antibodies specific for myosin heavy chain or other sarcomeric proteins (208). Differentiation potential varies significantly among different individuals, as demonstrated by the example of myosin staining shown in **Figure 2** (204).

Clearly future work will need to define more rapid, quantitative and reliable markers of myogenic stem cell able to predict the differentiation potential of MABs generated *in vitro* for tissue repair purposes. MABs are only in their first clinical application and future work will also hopefully allow to define whether markers of potency correlate with the capacity of these cells to regenerate muscle tissue *in vivo*. Finally, a better understanding of the different cell types and soluble players that together orchestrate the regeneration of a complex tissues like muscles will be required.

## 8 Summary and conclusions

ATMPs are promising drugs in many disease contexts. Potency assays for cell-based ATMPs are complex, often requiring several days or weeks to perform. Due to their complexity, they are not standardized and vary quite widely in different laboratories with little consensus about what specifications should applied for GMP release. Specifications are usually based on the preclinical experience of each laboratory. In some cases, potency assays are qualitative rather than quantitative, for example the capacity of stem cells to differentiate into different cell types. Another variable is whether potency assays are performed on fresh or frozen products or both. Cell numbers or costs may not allow the latter to be performed on each batch and may therefore rather be part of the process validation and of stability studies, as recently reviewed by our group (31).

It is important to establish for each cell type which potency assays should be performed for each use, either for batch release or for information only. Which assays to perform depends on the known predictive potential of the assays and whether the test is performed to demonstrate the fitness of the cell product or to predict *in vivo* efficacy. Indeed, the evidence that potency assays *in vitro* correlate with efficacy *in vivo* is still scarce and depends upon the specific ATMP, disease being treated as well as on the potency assays being performed. Correlations are particularly difficult to demonstrate when disease pathogenesis is complex, when multiple mechanisms of action of the ATMP may apply, and when uncertainty remains about the most important mechanism of action of the drug. Furthermore, patient specific factors (disease stage, burden or localization, tumor microenvironment, patient fitness, etc.), often play an important role in the clinical response to cell therapy, making correlation studies between potency assays *in vitro* and clinical response particularly arduous. Nonetheless potency assays and analyses of surrogate markers of potency are important to perform systematically during the development phase of an ATMP, using the most appropriate disease models *in vitro* and *in vivo* in order to try and define the mechanisms of action of ATMPs in different disease contexts and establish criteria for potency assays that may predict efficacy. Similarly, potency assays are important to include in clinical studies, so that possible correlations with efficacy may be performed. The feasibility of the potency assays obviously depends also on the size of the batches that are being produced and the complexity, cost and number of cells required to perform the assays. Thus, performing relevant *in vivo* potency assays in small animal models, for large ATMP batches used to treat hundreds of patients, is important, whereas, if possible, standardized simple assays, such as the identification of a biomarker or standardized functional assay *in vitro* are more suitable for small batches used to treat one or few patients.

Ideally surrogate markers of potency should be established, in order to provide fast, reproducible and more quantitative assays for potency definition. Unfortunately, very few markers of potency have yet been defined. The best example is the high expression of ΔNp63α in epithelial stem cells from different tissues, which is widely used since it correlates with tissue repair capacity. Nonetheless standardization of this marker should be aimed at for future clinical studies, as proposed by some groups (170). On the other end of the spectrum, some cells such as MSCs do not yet have any marker of potency or stemness or biological activity, which may define their capacity to mediate either immunosuppression or tissue repair. Nonetheless the recent technological progress for single cell analyses, such as scRNAseq and other “omics” analyses, should allow to identify in the near future more specific markers predicting the quality and efficacy for each ATMP and each specific use (206). Progress in this sense has already been made for example for CAR-T cell products as discussed above (76).

It is worth noting that, for many autologous or patient-dedicated ATMPs, it is important to speed up the delivery of the drug, either due to the gravity of the patients or to the fitness of the cellular product. For this reason shorter culture times and rapid administration of fresh rather than frozen products are approaches that are currently being introduced in several clinical contexts (209). In these cases, potency assays for release may not be feasible and may be more appropriately carried out only during the development and validation phase of the cell-based drugs, and subsequently for information only, rather than as a formal release assay.

## Author contributions

JG, MI have conceived and designed the work. all authors have contributed to the writing and critical revision of the manuscript, each for their expertise on specific ATMPs. MI, AB and GM have contributed to funding. All authors contributed to the article and approved the submitted version.
